# Risk of cellular or antibody-mediated rejection in pediatric kidney transplant recipients with BK polyomavirus replication—an international CERTAIN registry study

**DOI:** 10.1007/s00467-024-06501-7

**Published:** 2024-10-11

**Authors:** Alexander Fichtner, Jeremy Schmidt, Caner Süsal, Andrea Carraro, Jun Oh, Matthias Zirngibl, Sabine König, Isabella Guzzo, Lutz T. Weber, Atif Awan, Kai Krupka, Paul Schnitzler, Hans H. Hirsch, Burkhard Tönshoff, Britta Höcker

**Affiliations:** 1 Heidelberg University, Medical Faculty Heidelberg, Department of Pediatrics I, University Children’s Hospital, Im Neuenheimer Feld 430, Heidelberg, 69120 Germany; 2 Heidelberg University, Medical Faculty Heidelberg, Department of Transplantation Immunology, Institute of Immunology, Heidelberg, Germany; 3 Transplant Immunology Research Center of Excellence, Koç University, Istanbul, Turkey; 4 Pediatric Nephrology, Dialysis and Transplantation Unit, Department of Woman’s and Child’s Health, University Hospital of Padova, Via Giustiniani 3, 35128 Padua, Italy; 5 Department of Pediatric Nephrology, University Children’s Hospital, Martinistr. 52, 20246 Hamburg, Germany; 6 University Children’s Hospital, Hoppe-Seyler-Str. 1, 72076 Tübingen, Germany; 7 Department of General Pediatrics, University Children’s Hospital Münster, Waldeyerstraße 22, 48149 Münster, Germany; 8 Pediatric Nephrology and Renal Transplant Unit, Bambino Gesù Children’s Hospital–IRCCS, Piazza S. Onofrio 4, 00165 Rome, Italy; 9 Pediatric Nephrology, Children’s and Adolescents’ Hospital, University Hospital of Cologne, Faculty of Medicine, University of Cologne, Kerpener Street 62, 50937 Cologne, Germany; 10 Temple Street Children’s University Hospital, Dublin 1, Ireland; 11 Department of Infectious Diseases, Virology, Heidelberg University, Medical Faculty Heidelberg, Im Neuenheimer Feld 324, Heidelberg, 69120 Germany; 12 Transplantation & Clinical Virology, Department Biomedicine, University of Basel, Petersplatz 10, 4009 Basel, Switzerland; 13 Infectious Diseases & Hospital Epidemiology, University Hospital Basel, Petersgraben 4, 4031 Basel, Switzerland

**Keywords:** Pediatric kidney transplantation, Donor-specific antibodies, BK polyomavirus-associated nephropathy, Kidney transplant rejection

## Abstract

**Background:**

In kidney transplant recipients (KTR), BK polyomavirus-associated nephropathy (BKPyVAN) is a major cause of graft loss. To facilitate the clearance of BKPyV-DNAemia, reduction of immunosuppression is currently the treatment of choice but may increase the risk of graft rejection.

**Methods:**

This international CERTAIN study was designed to determine the risk of alloimmune response and graft dysfunction associated with immunosuppression reduction for BKPyV treatment in 195 pediatric KTR.

**Results:**

BKPyV-DNAemia was associated with a more than twofold increased risk of late T cell-mediated rejection (TCMR) (HR 2.22, *p* = 0.024), of de novo donor-specific HLA antibodies (dnDSA) and/or antibody-mediated rejection (ABMR) (HR 2.64, *p* = 0.002), and of graft function deterioration (HR 2.73, *p* = 0.001). Additional independent risk factors for dnDSA/ABMR development were a higher HLA mismatch (HR 2.72, *p* = 0.006) and re-transplantation (HR 6.40, *p* = 0.000). Other independent predictors of graft function deterioration were TCMR (HR 3.98, *p* = 0.003), higher donor age (HR 1.03, *p* = 0.020), and re-transplantation (HR 3.56, *p* = 0.013).

**Conclusions:**

These data indicate that reduction of immunosuppression for BKPyV-DNAemia management is associated with increased alloimmune response in pediatric KTR. Therefore, regular dnDSA screening and close monitoring of graft function in case of BKPyV-DNAemia followed by subsequent reduction of immunosuppressive therapy are recommended.

**Graphical sbstract:**

**Figure:**
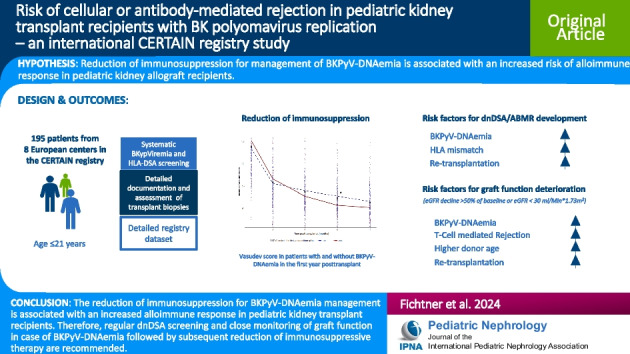
A higher resolution version of the Graphical abstract is available as Supplementary information

**Supplementary Information:**

The online version contains supplementary material available at 10.1007/s00467-024-06501-7.

## Introduction

In kidney transplant recipients, reactivation of BK polyomavirus (BKPyV) replication is a common consequence of immunosuppressive therapy, which can progress to BKPyV-associated nephropathy (BKPyVAN), and ultimately to graft loss [1]. Additionally, primary BKPyV infection can occur in BKPyV-naïve pediatric kidney transplant patients who lack BKPyV-specific cellular and humoral immunity [2, 3]. The rate of BKPyV-DNAemia is highest (33%) in the first post-transplant year. However, about 10% of pediatric patients do not develop BKPyV-DNAemia until only after the second post-transplant year [4]. High levels of BKPyV-DNAemia are also more common in pediatric patients than in adults. According to the North American Pediatric Renal Trials Collaborative Studies (NAPRTCS), 24% of pediatric kidney allograft recipients with BKPyVAN experience graft loss at an average of 24 months after diagnosis [5].

To date, antiviral agents with BKPyV-specific activity are lacking. Adjunctive therapies, including leflunomide, fluoroquinolones, and IVIG administration, have been evaluated in small studies in adult patients, with variable response rates [5–10]. Therefore, reduction of immunosuppression is currently the only effective strategy to reduce BKPyV-DNAemia and thus to prevent progression to BKPyVAN [1]. However, measures to reduce immunosuppression are not standardized and vary among transplant centers. Reduction or discontinuation of antiproliferative agents or calcineurin inhibitors are the most common approaches, but switching from a calcineurin inhibitor to a mammalian target of rapamycin (mTOR) inhibitor has also been performed to reduce BKPyV-DNAemia [11].

While reduction of immunosuppression is intended to facilitate BKPyV clearance by enhancing the patient’s BKPyV-specific cellular and humoral immune response, it may also carry the risk of an increased immune response to the allograft. A number of retrospective, single-center studies in adult kidney transplant recipients have addressed the potential relationship between BKPyV-DNAemia, reduction of immunosuppression, and the development of de novo HLA donor-specific antibodies (dnDSA) and/or transplant rejection, with mixed results [12–25]. Data in the pediatric kidney transplant population are scarce [26, 27].

The objective of this study was to determine the risk of alloimmune responses, namely T cell-mediated rejection (TCMR) and development of dnDSA and/or antibody-mediated rejection (ABMR), resulting from the reduction of immunosuppression for BKPyV-DNAemia management purposes in pediatric kidney allograft recipients. An additional objective was to evaluate risk factors for graft dysfunction in this patient population.

## Materials and methods

### Study design and patients

We performed a retrospective, international, multicenter, longitudinal cohort analysis of data reported to the Cooperative European Paediatric Renal Transplant Initiative (CERTAIN) registry (www.certain-registry.eu) [28, 29]. This registry allows an in-depth characterization of specific patient cohorts, due to its detailed and comprehensive data collection. Specific and additional data collection for this analysis was performed according to a defined protocol. Eligible patients were pediatric kidney allograft recipients (i) aged ≤ 21 years at the time of transplantation; (ii) with a complete and validated data set; (iii) with plasma BKPyV surveillance by nucleic acid testing (NAT) at least twice during the first year post-transplant and yearly thereafter; (iv) with dnDSA surveillance at least once a year, and (v) with at least 1 year of follow-up post-transplant. All diagnostic investigations for BKPyV or dnDSA events had to be documented including those that were negative. Study centers were asked to enroll all patients who met the above-mentioned inclusion criteria in order to avoid a selection bias. A total of 195 pediatric patients from 8 European pediatric renal transplant centers, who underwent kidney transplantation between 2007 and 2019, were included in the study. Patient and transplant characteristics are shown in Table 1. A subgroup analysis was performed in patients at low immunologic risk (*n* = 154), who were defined as those without re-transplantation, preformed HLA donor-specific antibodies, or desensitization treatment (induction therapy with rituximab or thymoglobulin, plasmapheresis, immunoadsorption). Data were collected on day 0 before transplantation, at months 1, 3, 6, 9, and 12 post-transplant, and every 6 months thereafter. Written informed consent was obtained from all parents/guardians to participate in the registry, with assent from patients when appropriate for their age. The CERTAIN registry was approved by the ethics committee of each contributing center and is fully compliant with the principles of the Declaration of Helsinki and Good Clinical Practice guidelines. The study was designed, analyzed, and reported according to the STROBE guidelines (https://www.strobe-statement.org).

**Table 1:** Baseline and transplant characteristics of the entire patient cohort

Parameter	Entire cohort (*n* = 195)	BKPyV-DNAemia (*n* = 59)	No BKPyV-DNAemia (*n* = 136)	*P* value
Age at KTx, years (median, IQR)	11.2 (5.2–14.7)	8.25 (4.4–13.4)	12.0 (5.79–15.1)	**0.011**
Male gender, *n* (%)	114 (58.5)	39 (66.1)	75 (55.2)	0.205
Caucasian, *n* (%)	184 (94.4)	55 (93.2)	129 (94.9)	0.908
> 1 KTx, *n* (%)^a^	17 (8.7)	3 (5.1)	14 (10.3)	0.364
Living donation, *n* (%)	63 (32.3)	18 (30.5)	45 (33.1)	0.852
Donor age, years (median, IQR)	36.0 (14–45)	28.0 (13.5–43.0)	38.0 (14.0–46.0)	0.314
Cold ischemia time, hours (median, IQR)	10.0 (3.0–14.3)	10.0 (2.9–15.2)	10.0 (3.0–14.1)	0.687
HLA mismatch, *n* (mean ± SD)	2.6 ± 1.2	2.47 ± 1.25	2.61 ± 1.17	0.472
HLA-DR mismatch, *n* (mean ± SD)	0.82 ± 0.61	0.86 ± 0.66	0.79 ± 0.60	0.513
Delayed graft function, *n* (%)	15 (7.7)	3 (5.1)	12 (8.9)	0.544
Preformed HLA donor-specific antibodies (DSA)	23 (11.8)	6 (10.2)	17 (12.5)	0.810
Initial eGFR, mL/min·1.73 m^2^ (mean ± SD)^b^	77.0 ± 29.9	77.5 ± 24.3	76.8 ± 32.1	0.462
Initial immunosuppressive regimen^c^				
Plasmapheresis, *n* (%)	8 (4.1)	0	8 (5.9)	0.109
Immunoadsorption, *n* (%)	3 (1.6)	0	3 (2.2)	0.555
Rituximab, *n* (%)	5 (2.6)	1 (1.7)	4 (2.9)	1.000
Thymoglobulin, *n* (%)	10 (5.1)	3 (5.1)	7 (5.2)	1.000
IL-2R antibody induction, *n* (%)	64 (32.8)	17 (28.8)	47 (34.6)	0.536
Maintenance immunosuppression				
TAC, *n* (%)	167 (85.6)	54 (91.5)	113 (83.1)	0.187
CSA, *n* (%)	28 (14.4)	5 (8.5)	23 (16.9)	0.187
MMF, *n* (%)	173 (88.7)	50 (84.8)	123 (90.4)	0.364
EVR, *n* (%)	12 (6.2)	5 (8.5)	7 (5.2)	0.517
AZA, *n* (%)	10 (5.1)	4 (6.8)	6 (4.4)	0.494
Steroids, *n* (%)	193 (99.0)	58 (98.3)	135 (99.3)	1.000
TAC/MMF, *n* (%)	150 (76.9)	47 (79.7)	103 (75.7)	0.680
TAC/EVR, *n* (%)	8 (4.1)	3 (5.1)	5 (3.7)	0.700
TAC/AZA, *n* (%)	9 (4.6)	4 (6.8)	5 (3.7)	0.458
CSA/MMF, *n* (%)	23 (11.8)	3 (5.1)	20 (14.7)	0.095
CSA/EVR, *n* (%)	4 (2.1)	2 (3.4)	2 (1.5)	0.586
CSA/AZA, *n* (%)	1 (0.5)	0	1 (0.7)	1.000
Pediatric Vasudev score (mean ± SD)^d,e^	14.6 ± 5.1	15.3 ± 5.4	14.4 ± 4.9	0.210

*BKPyV* BK polyomavirus, *KTx* kidney transplantation, *HLA* human leukocyte antigen, *DSA* donor-specific antibodies, *eGFR* estimated glomerular filtration rate, *IL-2R* interleukin 2 receptor, *TAC* tacrolimus, *CSA* cyclosporine microemulsion, *MMF* mycophenolate mofetil, *EVR* everolimus, *AZA* azathioprine

^a^For patients having undergone more than one kidney transplantation, only the most recent one was evaluated

^b^Defined as eGFR at 30 days of post-transplant. eGFR was calculated according to the revised Schwartz formula: eGFR = (mL/min·1.73 m^2^) = 0.413 × [height (cm)/serum creatinine (mg/dL)]

^c^Until day 30 of post-transplant

^d^Defined in ref. 4, 31

^e^At 30 days of post-transplant

### BKPyV replication and BKPyVAN

Assessment of BKPyV-DNAemia and BKPyVAN was performed according to center practice. Quantitative nucleic acid testing (QNAT) was performed using commercially available assays. Patients with quantifiable BKPyV-DNA detection in the blood (above the lower limit of quantification) were regarded as BKPyV-DNAemia positive. High-level BKPyV-DNAemia was defined as > 10^4^ copies/mL according to international guidelines [1]. Biopsy-proven BKPyVAN was diagnosed by the local pathologist at each center according to the respective most recent Banff classification [30].

### Immunosuppressive regimen and treatment of BKPyV replication and BKPyVAN

Immunosuppressive therapy typically included a calcineurin inhibitor (CNI), namely tacrolimus (TAC) or cyclosporine microemulsion (CSA), an antiproliferative agent, namely mycophenolate mofetil (MMF), azathioprine (AZA) or mammalian target of rapamycin inhibitor (mTORi) such as everolimus (EVR) or sirolimus (SIR), and glucocorticoids (Table 1). The net state of immunosuppression was calculated for each patient using a semiquantitative immunosuppression score called the modified Vasudev score (Supplemental Table 1) [4, 31]. Treatment of BKPyV-DNAemia and BKPyVAN was performed at the discretion of the treating physician, including reduction of immunosuppressive medication and adjunctive therapy (fluoroquinolones, cidofovir, intravenous immunoglobulins [IVIG]).

### Donor-specific HLA antibodies

Patient sera were tested for HLA antibodies and their specificities according to center practice, at least once annually and at the time of an indication biopsy. LABScreen Luminex kits from One Lambda (Canoga Park, CA, USA) were used. Irrespective of the individual definition of DSA-positivity in the participating centers, a mean fluorescence intensity (MFI) of ≥ 1400 was defined as a universal cut-off value indicating DSA positivity in this multicenter setting [32]. Any new antibody was considered de novo whereas an antibody present at the allelic level before and after transplantation was considered preformed.

### Graft function and rejection episodes

Graft function was assessed as estimated glomerular filtration rate (eGFR) which was calculated according to the revised Schwartz formula: eGFR = (mL/min per 1.73 m^2^) = 0.413 × [height (cm)/serum creatinine (mg/dL)] [33, 34]. Graft function deterioration was defined as an eGFR < 30 mL/min·1.73 m^2^ or an eGFR decline ≥ 50% of baseline (at month 1 post-transplant). Biopsy-proven rejection episodes (T cell-mediated rejection (TCMR) and or antibody-mediated rejection (ABMR)) and other histopathologic findings were diagnosed by the local pathologist at each center according to the respective most recent Banff classification (2019) [30]. In this study, all biopsies were indication biopsies. Histopathological signs of TCMR in addition to detection of SV40Ag were not diagnosed as TCMR but BKPyVAN. In the absence of SV40Ag detection, histopathological findings of TCMR were diagnosed as TCMR. Since the issue of TCMR diagnosis under ongoing BKPyV-DNAemia can be difficult and is currently discussed controversially in the literature, we performed two analyses, one including and a second excluding TCMR episodes during BKPyV-DNAemia.

### Statistical analyses

Data were analyzed using PASW (SPSS) Statistics 27.0 and R 3.62. Results for continuous variables are given as mean ± standard deviation or median (interquartile range), depending on whether they were normally distributed or not. Categorical parameters are expressed as the number and percentage of patients. The Shapiro–Wilk test was used to assess whether the data were normally distributed. After testing the proportional hazards assumption, Cox regression models with time-dependent variables were used to examine the association of factors with the development of (i) TCMR, (ii) dnDSA and/or ABMR, and (iii) eGFR loss. Covariates included recipient and donor age, sex, ethnicity, cold ischemia time, donor type, re-transplantation, HLA mismatch, delayed graft function (DGF), desensitization treatment, panel reactive antibody level, preformed DSA, level of BKPyV viral load, persistence of BKPyV viral load, clearance of BKPyV and immunosuppressive medication. Factors with a *P* value < 0.20 according to univariate analysis were included in the multivariable model. Furthermore, an alternative approach was employed for the analysis of data in order to reinforce the results of the time-dependent Cox regression analysis (Kaplan–Meier analysis with a fixed landmark at 12 months post-transplant). Patients who were lost to follow-up or did not reach an observation period of 5 years post-transplant were censored as having no event. The course of the modified Vasudev score during the first year post-transplant was modelled with a mixed linear model and illustrated by segmented linear regression plots. A two-tailed *P* value < 0.05 was considered statistically significant. All analyses were exploratory, not confirmatory.

## Results

### Patient cohort

**Fig. 1:**
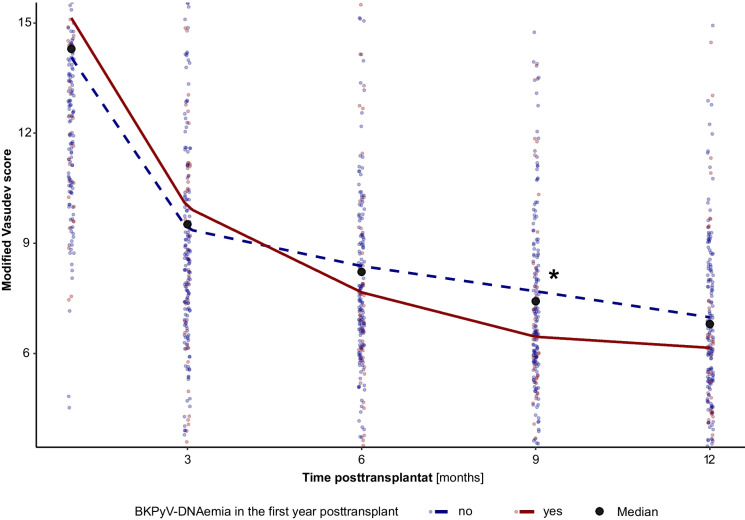
Segmented linear regression of the modified Vasudev score in patients with and without BKPyV-DNAemia (1-year data). Asterisk (*) marks the point of time with a significant difference in the modified Vasudev score between the two groups (p = 0.022). The slope of the Vasudev score decline between months three and six was also found to be significantly different (p = 0.013). The plot has been magnified in order to enhance the visibility (comparable full scale in Figure S1)

**Fig. 2:**
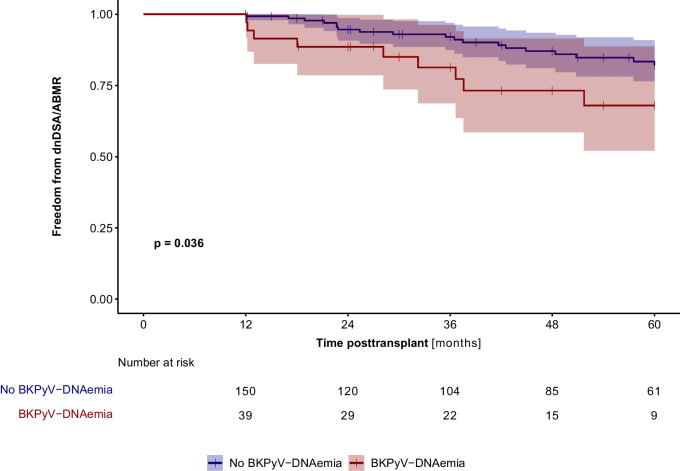
Freedom from de novo HLA-DSA (dnDSA) positivity and/or antibody-mediated rejection (ABMR) in patients (entire cohort, n = 195) with (red line) and without (blue line) BKPyV-DNAemia during the first 12 months of post-transplant (landmark analysis). BKPyV-DNAemia was significantly associated with the development of dnDSA/ABMR (p = 0.036)

A total of 195 pediatric patients who underwent kidney transplantation between 2007 and 2019, were included in this study. Patient demographics, clinical parameters, and initial immunosuppressive regimen are shown in Table 1. One-year patient and graft survival rates were 100%; at 5 years post-transplant, patient and graft survival rates (uncensored for death) were 98.9% (92/93) and 95.7% (89/93), respectively (Figs. 1 and 2).

### BKPyV-DNAemia

One-third of patients (59/195, 30.3%) developed BKPyV-DNAemia during the 5-year study period, with a median onset at 5.6 (IQR, 2.5–10.2) months post-transplant; the majority of these patients (45/59, 76.3%) had BKPyV-DNAemia already within the first year post-transplant. The overall rate of BKPyV-DNAemia during the first post-transplant year was 23.1% (45/195). High-level BKPyV-DNAemia (> 10^4^ copies/mL) was observed in 35/195 (17.9%) recipients. Thirty-five of 59 patients with BKPyV-DNAemia underwent a transplant kidney biopsy. Biopsy-proven BKPyVAN was diagnosed in 13/195 (6.7%) patients and in (13/59) 22.0% of patients with BKPyV-DNAemia. All patients with biopsy-proven BKPyVAN showed high-level BKPyV-DNAemia. The median time from BKPyV-DNAemia to BKPyVAN was 8.13 months (4.01–11.4). Treatment of BKPyV-DNAemia and BKPyVAN included CNI dose reduction or discontinuation (42/59, 71.2%), MMF dose reduction or withdrawal (20/59, 33.9%), switching from TAC to CSA (9/59, 15.3%), switching from MMF to EVR (7/59, 11.9%), or a combination of these measures. The net state of immunosuppression, as expressed by the modified Vasudev score, is depicted in Fig. 1 and Supplemental Figure S1 (1-year data). As can be seen, the reduction in the total immunosuppressive burden was significantly more pronounced in patients with BKPyV-DNAemia than the standard reduction of immunosuppression after transplantation in patients without BKPyV-DNAemia. Adjunctive therapy included administration of fluoroquinolones (6/59, 10.2%), cidofovir (8/59, 13.4%), and IVIG (7/59, 11.9%). A total of 47 (79.7%) patients cleared BKPyV-DNAemia during the study period. Among those with high-level BKPyV-DNAemia, 26 (74.4%) showed BKPyV-DNAemia clearance including four patients (30.8%) with BKPyVAN. The median time to BKPyV-DNAemia clearance was 4.17 months (2.12–18.6).

### T cell-mediated rejection

In the first year post-transplant, 56 (28.7%) patients developed TCMR. Late TCMR (more than 12 months of post-transplant) was observed in 34 patients during the second and fifth year post-transplant. The time from transplantation to TCMR development was significantly (*p* = 0.001) longer in patients with BKPyV-DNAemia (median 18.4 months, IQR 4.69–30.7) than in BKPyV-DNAemia-free (median 3.70, IQR 0.56–11.31) patients. The median time from onset of BKPyV-DNAemia to TCMR was 12.3 months (5.71–18.3). According to univariate Cox regression with time-dependent covariate analysis of TCMR development, BKPyV-DNAemia was significantly (HR 2.22, *p* = 0.024) associated with an increased risk of late TCMR (> 12 months post-transplant), presumably due to reduced immunosuppression chosen as treatment for BKPyV-DNAemia. At 9 months post-transplant, the overall immunosuppression score (modified Vasudev score) was indeed significantly lower in patients with BKPyV-DNAemia than in BKPyV-free transplant recipients, namely 6.86 ± 2.99 *vs.* 7.73 ± 3.03 (*p* = 0.0479), whereas the initial immunosuppression score at 30 days post-transplant was comparable between the groups (modified Vasudev score, 15.32 ± 5.41 *vs.* 14.35 ± 4.93, *p* = 0.210). A detailed course of the modified Vasudev score in the first year post-transplant is provided in Fig. 1﻿ and Supplemental Figure S1.

In this study, ten patients were treated for biopsy-confirmed TCMR (being negative for SV40Ag) diagnosed during ongoing BKPyV-DNAemia. If these patients were excluded from the analysis, no significant association of BKPyV-DNAemia and late TCMR was observed. No independent risk factor for the development of TCMR was identified (data not shown).

### De novo donor-specific HLA antibodies and antibody-mediated rejection

Forty-nine of 195 patients (25.1%) developed dnDSA during 5 years of follow-up, with a median onset at 12.9 (IQR, 3.6–33.6) months post-transplant. Twenty-two (11.3%) recipients developed dnDSA within the first year post-transplant; 14.4% of patients developed class II dnDSA only, 7.2% had both class I and II dnDSA, and 3.6% had solely class I dnDSA. According to multivariable Cox regression analysis with time-dependent covariates, independent risk factors for the development of dnDSA were BKPyV-DNAemia (HR 2.61, *p* = 0.004), a higher HLA mismatch (HR 3.13, *p* = 0.005), and re-transplantation (HR 4.87, *p* = 0.000) (Table 2). Fifty-six of 195 patients (28.7%) developed dnDSA and/or ABMR during the observation period. The median time from onset of BKPyV-DNAemia to the first detection of dnDSA/ABMR was 15.6 months (8.39–29.4). The rate of dnDNA and/or ABMR was significantly higher in patients with BKPyV-DNAemia (24/59, 40.7%) than in those without (32/136, 23.5%, *p* = 0.024). In addition, patients with BKPyV-DNAemia in the first 12 months of post-transplant developed dnDSA/ABMR significantly (*p* = 0.036) earlier than BKPyV-DNAemia-free patients (landmark analysis Kaplan–Meier, Fig. 2). According to multivariable Cox regression analysis with time-dependent covariates, independent risk factors for the development of dnDSA and/or ABMR were BKPyV-DNAemia (HR 2.64, *p* = 0.002), a higher HLA mismatch (HR 2.72, *p* = 0.006), and re-transplantation (HR 6.40, *p* = 0.000) (Table 2).

**Table 2:** Risk factors analysis

	Unadjusted HR (95% CI)	*P* value	Adjusted HR (95% CI)	*P* value
Risk factors for developing dnDSA and/or ABMR				
Entire cohort				
BKPyV-DNAemia^#^	2.24 (1.21–4.18)	0.011	2.64 (1.41–4.94)	0.002
Higher HLA mismatch	2.62 (1.32–5.20)	0.005	2.72 (1.34–5.56)	0.006
Re-transplantation	5.75 (2.99–11.1)	0.000	6.40 (3.30–12.4)	0.000
Low immunologic-risk patients				
BKPyV-DNAemia^#^	2.83 (1.35–5.96)	0.006	2.79 (1.33–5.87)	0.007
Higher HLA-DR mismatch	2.01 (1.15– 3.52)	0.015	1.98 (1.14–3.46)	0.016
Risk factors for eGFR loss				
Entire cohort				
BKPyV-DNAemia^#^	2.45 (1.20–5.02)	0.015	2.73 (1.22–6.07)	0.014
TCMR^#^	4.94 (2.02–12.1)	0.000	3.98 (1.61–9.80)	0.003
Higher donor age (years)	1.04 (1.01–1.07)	0.008	1.03 (1.01–1.06)	0.020
Re-transplantation	4.38 (1.76–10.9)	0.001	3.56 (1.30–9.75)	0.013
dnDSA/ABMR^#^	2.37 (1.16–4.87)	0.018		
Low immunologic-risk patients				
BKPyV-DNAemia^#^	4.47 (1.78–11.2)	0.001	4.51 (1.79–11.4)	0.001
TCMR^#^	5.12 (1.71–15.3)	0.004	5.14 (1.72–15.4)	0.004

*BKPyV* BK polyomavirus, *dnDSA *de novo donor-specific antibodies, *ABMR* antibody-mediated rejection, *HLA* human leukocyte antigen, *eGFR* estimated glomerular filtration rate, *TCMR* T cell-mediated rejection

^#^Variable included as time-dependent variable in the Cox regression model

### Graft function

Graft function deterioration, defined as an eGFR < 30 mL/min·1.73 m^2^ or an eGFR decline ≥ 50% of baseline, occurred in 30 (15.4%) patients during the observation period. The incidence of this event (eGFR < 30 mL/min·1.73 m^2^ or an eGFR decline ≥ 50% of baseline) was significantly (*p* = 0.019) higher in pediatric kidney allograft recipients with BKPyV-DNAemia (15/59, 25.4%) than in those without (15/136, 11.0%). The median time from onset of BKPyV-DNAemia to graft function deterioration was 22.0 months (10.1–51.1). According to univariate Cox regression analysis with time-dependent covariates, BKPyV-DNAemia, TCMR, dnDSA/ABMR, older donor age, and re-transplantation were associated with eGFR loss. However, multivariable modeling revealed only BKPyV-DNAemia (HR 2.73, *p* = 0.003), TCMR (HR 3.87, *p* = 0.003), older donor age (HR 1.03, *p* = 0.020), and re-transplantation (HR 3.56, *p* = 0.013) as independent risk factors for eGFR loss in this patient population (Table 2﻿ and Supplemental Table S2).

### Subgroup analysis of patients with low immunologic risk

One hundred fifty-four of 195 patients (79.0%) had a low immunologic risk profile. Patient characteristics and clinical parameters are shown in Table 2. Approximately one-third of patients received a kidney from a living donor. Delayed graft function occurred in 5.8% of patients. The majority of patients received an initial immunosuppressive regimen consisting of TAC, MMF, and steroids; and 39.0% received IL-2R antibody induction therapy (Table ﻿3).

**Table 3:** Baseline and transplant characteristics of patients with low immunologic risk

Parameter	Entire cohort (*n* = 154)	BKPyV-DNAemia (*n* = 48)	No BKPyV-DNAemia (*n* = 106)	*P* value
Age at KTx, years (median, IQR)	10.7 (4.9–14.3)	8.2 (3.5–13.0)	11.5 (5.6–14.5)	**0.037**
Male gender, *n* (%)	93 (60.4)	34 (70.8)	59 (55.7)	0.206
Caucasian, *n* (%)	144 (93.5)	45 (93.8)	99 (93.4)	0.737
Living donation, *n* (%)	54 (35.1)	17 (35.4)	37 (34.9)	0.868
Donor age, years (median, IQR)	36.0 (13.8–44.3)	30.0 (14.8–43.5)	36.5 (13.3–44.0)	0.884
Cold ischemia time, hours (median, IQR)	10.0 (3.0–14.1)	8.7 (2.3–15.2)	10.0 (3.0–13.6)	0.935
HLA mismatch, *n* (mean ± SD)	2.50 ± 1.2	2.29 ± 1.25	2.58 ± 1.24	0.191
HLA-DR mismatch, *n* (mean ± SD)	0.81 ± 0.63	0.81 ± 0.67	0.80 ± 0.62	0.976
Delayed graft function, n (%)	9 (5.8)	2 (4.17)	7 (6.60)	0.560
Initial eGFR, mL/min·1.73 m^2^ (mean ± SD)^a^	78.5 ± 30.2	79.36 ± 25.5	78.10 ± 32.6	0.457
Initial immunosuppressive regimen^b^				
IL-2R antibody induction, *n* (%)	60 (39.0)	17 (35.4)	43 (40.6)	0.536
Maintenance immunosuppression				
TAC, *n* (%)	129 (83.8)	43 (89.6)	86 (81.1)	0.187
CSA, *n* (%)	25 (16.2)	5 (10.4)	20 (18.9)	0.187
MMF, *n* (%)	136 (88.3)	41 (85.4)	95 (89.6)	0.364
EVR, *n* (%)	10 (6.5)	4 (8.3)	6 (5.7)	0.518
AZA, *n* (%)	8 (5.2)	3 (6.3)	5 (4.7)	0.494
Steroids, *n* (%)	153 (99.4)	47 (97.9)	106 (100)	0.515
TAC/MMF, *n* (%)	115 (74.7)	38 (79.2)	77 (72.6)	0.680
TAC/EVR, *n* (%)	7 (4.5)	2 (4.2)	5 (4.7)	0.700
TAC/AZA, *n* (%)	7 (4.5)	4 (6.3)	4 (3.8)	0.459
CSA/MMF, *n* (%)	21 (13.6)	3 (6.3)	18 (17.0)	0.095
CSA/EVR, *n* (%)	3 (1.9)	2 (4.17)	1 (0.9)	0.586
CSA/AZA, *n* (%)	1 (0.6)	0	1 (0.9)	1.000
Pediatric Vasudev score (mean ± SD)^c,d^	14.4 ± 5.0	15.0 ± 5.3	14.1 ± 4.8	0.329

*BKPyV* BK polyomavirus, *KTx* kidney transplantation, *HLA* human leukocyte antigen, *eGFR* estimated glomerular filtration rate, *IL-2R* interleukin 2 receptor, *TAC* tacrolimus, *CSA* cyclosporine microemulsion, *MMF* mycophenolate mofetil, *EVR* everolimus, *AZA* azathioprine

^a^Defined as eGFR at 30 days of post-transplant. eGFR was calculated according to the revised Schwartz formula: eGFR = (mL/min·1.73 m^2^) = 0.413 × [height (cm)/serum creatinine (mg/dL)]

^b^Until day 30 of post-transplant

^c^Defined in ref. 4, 31

^d^At 30 days of post-transplant

One-third of patients (48/154, 31.2%) developed BKPyV-DNAemia over the 5-year study period, with a median onset at 5.5 (IQR 2.6–9.6) months after transplantation. The rate of patients with TCMR (including treated borderline rejection) was 26.0% (40/154) in the first post-transplant year. Late TCMR occurred in 27 patients during the second and fifth years post-transplant. BKPyV-DNAemia tended to be associated (HR 2.08, *p* = 0.024) with an increased risk of late TCMR (> 12 months of post-transplant). No independent risk factor for the development of late TCMR could be identified in patients with low immunologic risk (data not shown).

BKPyV-DNAemia (HR 2.64, p = 0.012) and a higher HLA mismatch (HR 2.46, *p* = 0.044) were independent risk factors for the development of dnDSA. Thirty-one patients (20.1%) of 154 patients developed dnDSA and/or ABMR during the observation period. The rate of dnDNA and/or ABMR was significantly higher in pediatric kidney allograft recipients with BKPyV-DNAemia (16/48, 33.3%) than in those without (15/106, 14.2%, *p* = 0.011). In addition, patients with BKPyV-DNAemia developed dnDSA/ABMR significantly (*p* = 0.010) earlier than patients without BKPyV-DNAemia during the first 12 months of post-transplant (Fig. ﻿3). Independent risk factors for the development of dnDSA and/or ABMR in low immunologic-risk patients were BKPyV-DNAemia (HR 2.79, *p* = 0.007) and a higher HLA DR mismatch (HR 1.98, *p* = 0.016) (Table ﻿2).

**Fig. 3:**
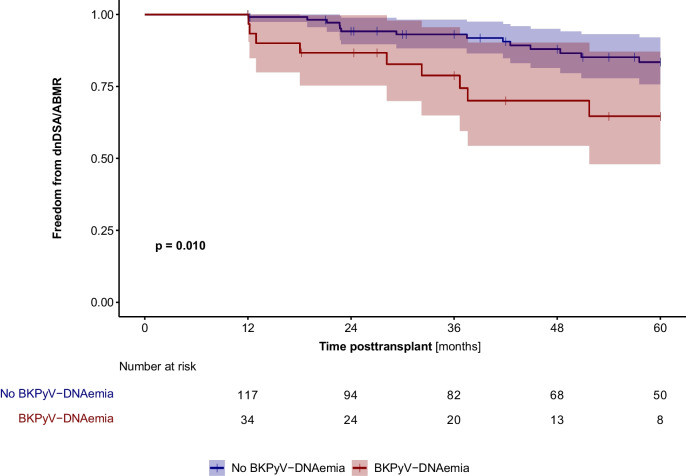
Freedom from de novo DSA (dnDSA) positivity and/or antibody-mediated rejection (ABMR) in low-risk patients (n = 154) with (red line) and without (blue line) BKPyV-DNAemia during the first 12 months of post-transplant (landmark analysis). BKPyV-DNAemia was significantly associated with the development of dnDSA/ABMR (p = 0.010)

Graft function deterioration, defined as an eGFR < 30 mL/min·1.73 m^2^ or an eGFR decline ≥ 50% of baseline, occurred in 20 (13.0%) patients during the 5-year follow-up period. The incidence of this event (eGFR < 30 mL/min·1.73 m^2^ or an eGFR decline ≥ 50% of baseline) was significantly (*p* = 0.005) higher in pediatric kidney allograft recipients with BKPyV-DNAemia (13/48, 27.1%) than in those without (7/106, 6.4%, *p* = 0.001). Independent risk factors for eGFR loss in low immunologic risk were BKPyV-DNAemia (HR 4.51, *p* = 0.001) and TCMR (HR 5.14, *p* = 0.004) (Table 2﻿). Excluding the ten patients with TCMR during BKPyV-DNAemia from the analysis had no impact on the pattern of risk factors for graft function deterioration (Supplemental Table S2).

## Discussion

The main findings of this international longitudinal cohort study of 195 pediatric kidney transplant recipients are the following: (i) BKPyV-DNAemia was significantly associated with a twofold increased risk of late TCMR, presumably due to reduced immunosuppressive therapy for management of BKPyV-DNAemia; (ii) patients with BKPyV-DNAemia developed dnDSA and/or ABMR more frequently and earlier than recipients without BKPyV-DNAemia (40.7% *vs*. 23.5%); (iii) BKPyV-DNAemia (HR 2.64), a higher HLA mismatch (HR 2.72), and re-transplantation (HR 6.40) were independent risk factors for dnDSA and /or ABMR; (iv) patients with BKPyV-DNAemia were significantly more likely to experience graft function deterioration than those without (25.4% *vs*. 11.0%); and (v) BKPyV-DNAemia (HR 2.73), TCMR (HR 3.87), a higher donor age (HR 1.03), and re-transplantation (HR 3.56) were found to be independent risk factors for eGFR loss. In patients with low-immunologic risk, (i) BKPyV-DNAemia was also associated with a higher rate and an earlier onset of dnDSA and/or ABMR (33.3% *vs*. 14.2%) and (ii) BKPyV-DNAemia (HR 2.74) and a higher HLA-DR mismatch (HR 1.98) were found to be independent risk factors for dnDSA and/or ABMR. In addition, (iii) graft function deterioration occurred significantly more often in patients with BKPyV-DNAemia than in those without (27.1% *vs*. 6.4%) and (iv) BKPyV-DNAemia (HR 4.51) and TCMR (HR 5.14) were significantly associated with a higher risk of eGFR loss in these patients.

This large multicenter study analyzed the risk of alloimmune responses (TCMR, dnDSA/ABMR) and graft function deterioration after reduced immunosuppression for BKPyV-DNAemia management in pediatric kidney allograft recipients. In this patient population, BKPyV-DNAemia was associated with a more than twofold risk of late (> 12 months of post-transplant) TCMR and dnDSA/ABMR development and up to 4.5-fold increased risk of graft function deterioration. Re-transplantation and a higher HLA mismatch were associated with an increased risk of dnDSA/ABMR development. Interestingly, in patients with a low immunologic risk, only the HLA *DR* mismatch but not total HLA mismatch, played a significant role in the development of dnDSA and/or ABMR.

The association of BKPyV-DNAemia and late TCMR is an interesting finding. However, one has to take into account that ten patients were diagnosed with TCMR (in the absence of SV40Ag) under ongoing BKPyV-DNAemia. The distinction between BKPyVAN and TCMR is challenging, and the diagnosis of TCMR during BKPyV-DNAemia cannot be diagnosed with certainty [35]. After excluding patients diagnosed with TCMR during BKPyV-DNAemia the association between BKPyV-DNAemia and late TCMR was no longer significant. However, the question of how to interpret interstitial nephritis in the context of BKPyV-DNAemia remains open. Two single-center, retrospective studies have addressed the issue of alloimmune response in pediatric kidney transplant recipients with BKPyV-DNAemia [26, 27, 36]. One study found no significant effect of BKPyV-DNAemia on the rate of graft rejection, patient, or graft survival. However, this study focused primarily on the analysis of risk factors for the development of BKPyV-DNAemia rather than on the outcome of BKPyV-DNAemia management. Rejection episodes were described only as “acute” or “chronic”, regardless of whether they were T cell- or antibody-mediated. No information was provided on dnDSA development or graft function deterioration [27]. Another small single-center study reported on 46 pediatric kidney allograft recipients, nine of whom had BKPyV-DNAemia. Two of these patients developed dnDSA, and one experienced ABMR. However, the small number of patients does not allow a valid statement on the risk of alloimmune reactions in this population [26].

In adult kidney allograft recipients, a number of single-center, retrospective studies have reported on the association between BKPyV-DNAemia or BKPyVAN and alloimmune responses to the graft. Our findings are consistent with most but not all of these studies in adults. Gras et al. conducted a single-center case–control study of 64 adult kidney transplant patients with biopsy-proven BKPyVAN and 64 controls [21]. The authors observed a higher rate of acute rejection episodes in recipients with BKPyVAN than in those without (19% *vs*. 3%, *p* = 0.003), a higher proportion of patients with dnDSA (MFI > 3000) (42% *vs*. 16%, *p* = 0.004), and a significantly worse kidney function in recipients with BKPyVAN. The results of that adult study support our findings in pediatric kidney transplant recipients but are limited to the fact that only patients with biopsy-proven BKPyVAN were included, but not those with BKPyV-DNAemia. Another single-center, retrospective study analyzed data from 174 adult kidney allograft recipients, 19 of whom had BKPyV-DNAemia [13]. Within 24 months after BKPyV-DNAemia detection, 79% of patients developed dnDSA, the majority of which occurred after BKPyV-DNAemia clearance. The authors concluded that post-transplant BKPyV-DNAemia and preemptive reduction of immunosuppression are associated with a higher risk of dnDSA, a finding that is consistent with our results.

In a single-center, retrospective study of 1019 adult kidney allograft recipients, Patel et al. sought to determine the association of BKPyV-DNAemia and dnDSA (MFI > 2000) [14]. One hundred and eighty-six patients had BKPyV-DNAemia, and 54 had progression to BKPyVAN. In all patients with BKPyV-DNAemia, immunosuppression was reduced to facilitate BKPyV clearance. The authors identified a higher HLA mismatch, a peak BKPyV viral load > 6 log_10_ copies/ml, and BKPyV clearance as independent risk factors for dnDSA development following BKPyV-DNAemia. These are interesting results, although it should be noted that the risk factor analysis was performed only in patients with BKPyV-DNAemia. Therefore, no statement can be made about the direct relationship between BKPyV-DNAemia and dnDSA development [14]. Another single-center, retrospective study examined risk factors for progression from low levels of BKPyV-DNAemia to adverse outcomes after reduction of immunosuppression for BKPyV management in 224 adult kidney allograft recipients [16]. Non-depleting induction at transplantation (HR 2.06), HLA mismatches > 3 (HR 2.27), and delayed graft function (HR: 4.14) were independently associated with the development of dnDSA and/or rejection in patients with BKyPVDNAemia. Because this study included only patients with BKPyV-DNAemia, no statement could be made about the relationship between BKPyV-DNAemia and dnDSA or rejection [16].

Sawinsky et al. analyzed the association between persistent BKPyV-DNAemia and dnDSA development (MFI > 800) in a single-center, retrospective study among 785 adult kidney or kidney/pancreas transplant recipients, 132 of whom developed BKPyV-DNAemia [15]. Persistent BKPyV-DNAemia did not adversely affect mid-term patient and graft survival in this cohort but was strongly associated with a twofold risk of class II dnDSA development, confirming our findings in pediatric kidney transplant recipients. Other independent risk factors for dnDSA in the adult cohort were panel reactive antibodies ≥ 30% and “acute rejection” [15]. Another single-center, retrospective study evaluated the relationship between BKPyVAN, isolated BKPyV-DNAemia, dnDSA (MFI > 1000), and the subsequent risk of ABMR in 904 adult kidney allograft recipients [17]. Thirty patients had BKPyV-DNAemia, and 43 patients had biopsy-proven BKPyVAN. BKPyVAN, but not BKPyV-DNAemia without histological findings, was a risk factor for dnDSA development in this study (HR 3.18, *p* = 0.08). In addition, BKPyVAN and dnDSA were significant predictors of ABMR and death-censored graft loss [17]. These results are partially consistent with our findings. However, in contrast to the adult study, BKPyV-DNAemia was significantly associated with dnDSA development in our pediatric population. When interpreting these results, it is important to note that in the adult study, only patients with a high BKPyV-DNAemia above 15,000 copies/ml were considered positive, whereas in our study all patients with BKPyV-DNAemia were considered positive. In addition, the authors noted that MMF was discontinued more frequently in their patients with BKPyVAN than in recipients with isolated high BKPyV-DNAemia, which may have contributed to their findings. Another explanation for the divergent results between the adult and our study may be the fact that the incidence of BKPyV-DNAemia is higher in pediatric than in adult allograft recipients, suggesting that a higher proportion of pediatric patients have an increased risk of uncontrolled BKPyV replication, which may have an impact on the risk of alloimmune reactions [4, 37].

A recently published single-center, retrospective case–control study analyzed data from 190 adult kidney allograft recipients with BKPyV-DNAemia and 396 controls without BKPyV-DNAemia [20]. BKPyV positivity was defined as BKPyV-DNAemia > 10,000 copies/ml or BKPyVAN. dnDSA rates were higher in patients with BKPyV-DNAemia, with the majority of dnDSA detected after reduction of immunosuppression as BKPyV management, and occurred earlier post-transplant than in controls. In addition, ABMR occurred earlier in BKPyV-positive patients, although the overall rates of ABMR were comparable between cases and controls [20]. The authors concluded that their data suggest an association between reduced immunosuppression and the development of dnDSA and/or rejection, and support close monitoring for dnDSA in patients with reduced immunosuppression due to BKPyV-DNAemia [20]. Graft function and graft survival data were not reported in this study. Another retrospective, single-center study analyzed the long-term outcome of 644 Swiss adult kidney allograft recipients including 105 patients with immunosuppression reduction for BKPyV-DNAemia management [18]. In these patients, in whom the CNI dose was reduced as a first step to manage BKPyV-DNAemia, predictors of adverse outcome were a high viral load and clinical rejection after BKPyV-DNAemia clearance. A risk factor analysis for adverse outcome in the overall patient cohort, including BKPyV-DNAemia as a cofactor, was not performed in this study. In this study, only endothelialitis and ABMR were considered alloimmune reactions; and no dnDSA measurement was performed [18]. As mentioned before, the diagnosis of TCMR during ongoing BKPyV-DNAemia remains challenging. The exact mechanism of BKPyV-DNAemia-associated alloimmune reactivity remains unclear. There may be a competing mechanism for alloimmune risk: besides reduced immunosuppression, interstitial nephritis associated with BKPyVAN may itself instigate alloreactivity. This would be associated with both increased immune cell trafficking to the allograft, and increased donor cellular debris/APCs trafficking to the secondary lymphoid organs during the period of active infection. Hence, further biomarkers are needed to interpret histopathological findings in the context of BKPyV-DNAemia. A number of reports detail the time course of inflammatory markers such as CXCL10 during BK viremia, which is actually higher than what is usually seen in rejection [38–40]. Persistence of CXCL10 expression following BKV infection resolution is also associated with subsequent rejection risk [40].

A further number of retrospective studies have reported on intermediate or long-term outcomes in adult kidney allograft recipients with BKPyV-DNAemia or BKPyVAN, with varying results [19, 22–25]. However, a direct comparison of their data with those of our pediatric study is difficult. Some of these studies lacked a control cohort without BKPyV-DNAemia, or dnDSA assessment. Others evaluated “viremia” as a potential risk factor for alloimmune reactions, but did not distinguish between different viruses such as BKPyV, JC polyomavirus, cytomegalovirus, or Epstein-Barr virus.

Our study has limitations, the most important of which is the retrospective study design, which is common to registry analyses in general. Another limitation is the lack of central laboratory viral load and dnDSA assessments. However, registries reflecting the real-world scenario with heterogeneous immunosuppressive regimens and treatment protocols are potentially suitable to provide a broader view of outcome and complications, especially in small cohorts such as pediatric kidney allograft recipients. In addition, large, multicenter cohort studies, such as our present one, allow for hypothesis generation and are complementary to prospective randomized trials. Another limitation is that we cannot draw conclusions about which immunosuppressive reduction strategy is best to treat BKPyV-DNAemia and about the risk associated with dose reduction or discontinuation of specific immunosuppressants, since in most patients a combination of different approaches was used. In conclusion, our study indicates that reduction of immunosuppression for BKPyV-DNAemia treatment is associated with an increased risk of alloimmune reactivity (late TCMR and dnDSA/ABMR) in the pediatric kidney allograft population. Additional risk factors for the development of dnDSA/ABMR were found to be a higher HLA mismatch and re-transplantation. Moreover, BKPyV-DNAemia and TCMR were found to be independent predictors of graft function deterioration in pediatric kidney allograft recipients. Our data suggest that regular dnDSA screening and close monitoring of graft function in pediatric patients with BKPyV-DNAemia followed by subsequent reduction of immunosuppression are recommended. Prospective, multicenter studies are needed to confirm our results and to elucidate the exact mechanism of BKPyV-DNAemia-associated alloimmune reactivity in pediatric kidney transplant recipients.

## Supplementary Information

Below is the link to the electronic supplementary material.

Graphical abstract (PPTX 169 KB)

Supplementary file2 (PDF 323 KB)

## Data Availability

The data that support the findings of this study are available from the corresponding author upon reasonable request.

## Abbreviations

ABMR: Antibody-mediated rejection
AZA: Azathioprine
BKPyV: BK polyomavirus
BKPyVAN: BK polyomavirus-associated nephropathy
CERTAIN: Cooperative European Paediatric Renal Transplant Initiative
CI: Confidence interval
CNI: Calcineurin inhibitor
CSA: Cyclosporine microemulsion
DGF: Delayed graft function
DNA: Deoxyribonucleic acid
dnDSA: *De novo* Donor-specific HLA antibodies
eGFR: Estimated glomerular filtration rate
EVR: Everolimus
HR: Hazard ratio
IVIG: Intravenous immunoglobulin
IL-2R: Interleukin 2 receptor
KTR: Kidney transplant recipient
MFI: Mean fluorescence intensity
MMF: Mycophenolate mofetil
mTORi: Mammalian target of rapamycin inhibitor
NAT: Nucleic acid testing
SIR: Sirolimus
TAC: Tacrolimus
TCMR: T cell-mediated rejection
