# Supplementary material for: Risk of cellular or antibody-mediated rejection in pediatric kidney transplant recipients with BK polyomavirus replication—an international CERTAIN registry study
Source: Pediatr Nephrol. 2024 Oct 11;40(3):835–48. doi: 10.1007/s00467-024-06501-7 (PMC11753334; doi:10.1007/s00467-024-06501-7)
Supplement: Supplementary file 1 — Graphical abstract (PPTX 169 KB) [file 467_2024_6501_MOESM1_ESM.pptx]

## Slide 1
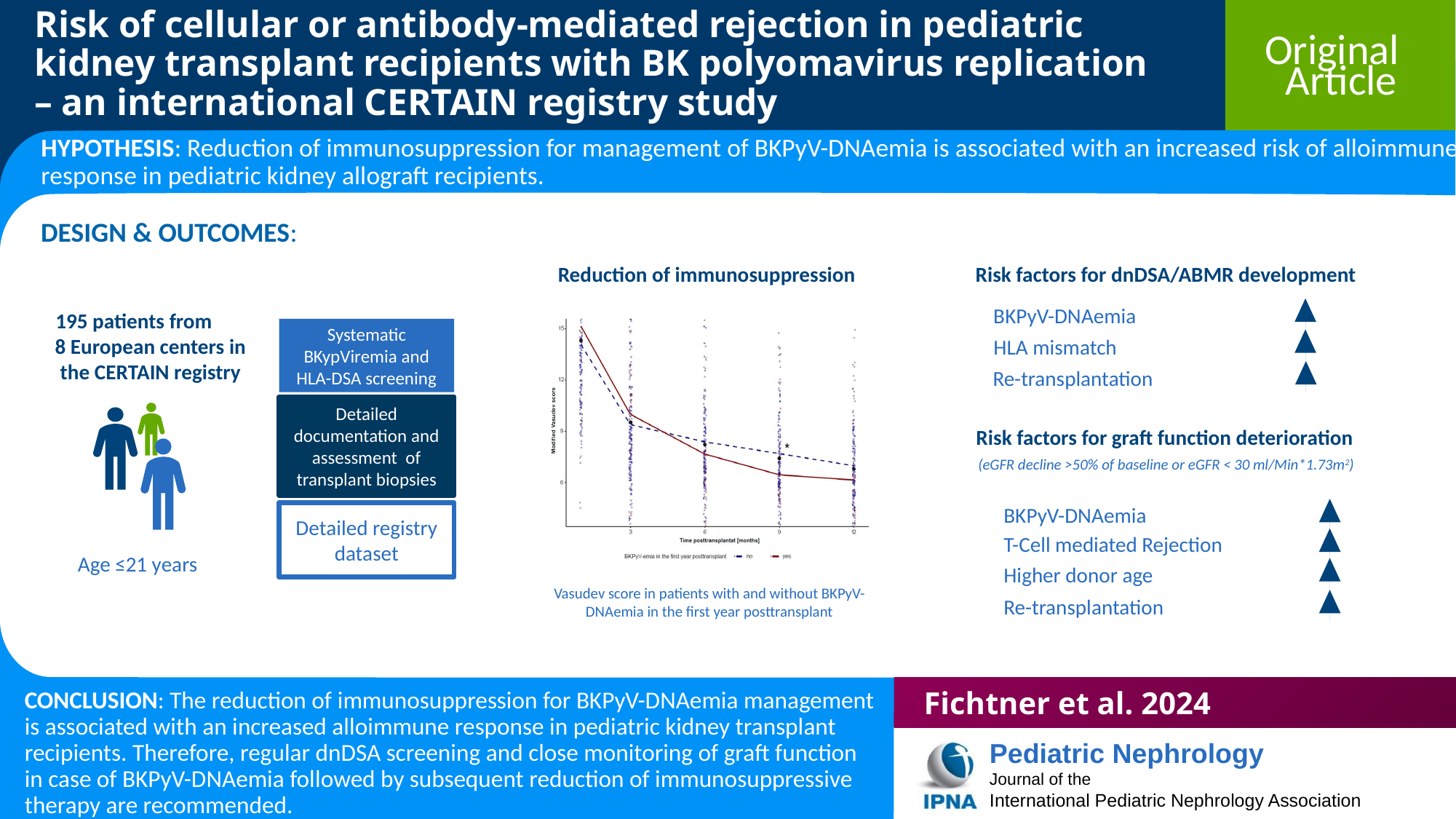

Risk of cellular or antibody-mediated rejection in pediatric kidney transplant recipients with BK polyomavirus replication
– an international CERTAIN registry study
HYPOTHESIS: Reduction of immunosuppression for management of BKPyV-DNAemia is associated with an increased risk of alloimmune response in pediatric kidney allograft recipients.
DESIGN & OUTCOMES:
Risk factors for dnDSA/ABMR development
Reduction of immunosuppression
BKPyV-DNAemia
195 patients from 8 European centers in the CERTAIN registry
Systematic BKypViremia and HLA-DSA screening
HLA mismatch
Re-transplantation
Detailed documentation and assessment of transplant biopsies
Risk factors for graft function deterioration
(eGFR decline >50% of baseline or eGFR < 30 ml/Min*1.73m2)
BKPyV-DNAemia
Detailed registry dataset
T-Cell mediated Rejection
Age ≤21 years
Higher donor age
Vasudev score in patients with and without BKPyV-DNAemia in the first year posttransplant
Re-transplantation
Fichtner et al. 2024
CONCLUSION: The reduction of immunosuppression for BKPyV-DNAemia management is associated with an increased alloimmune response in pediatric kidney transplant recipients. Therefore, regular dnDSA screening and close monitoring of graft function in case of BKPyV-DNAemia followed by subsequent reduction of immunosuppressive therapy are recommended.
