# Supplementary material for: Risk of cellular or antibody-mediated rejection in pediatric kidney transplant recipients with BK polyomavirus replication—an international CERTAIN registry study
Source: Pediatr Nephrol. 2024 Oct 11;40(3):835–48. doi: 10.1007/s00467-024-06501-7 (PMC11753334; doi:10.1007/s00467-024-06501-7)
Supplement: Supplementary file 2 — Supplementary file2 (PDF 323 KB) [file 467_2024_6501_MOESM2_ESM.pdf]

Supplemental Information

Supplemental Figure S 1 Individual courses of modified Vasudev score in patients with and without BKPyV-DNAemia (1-year data)

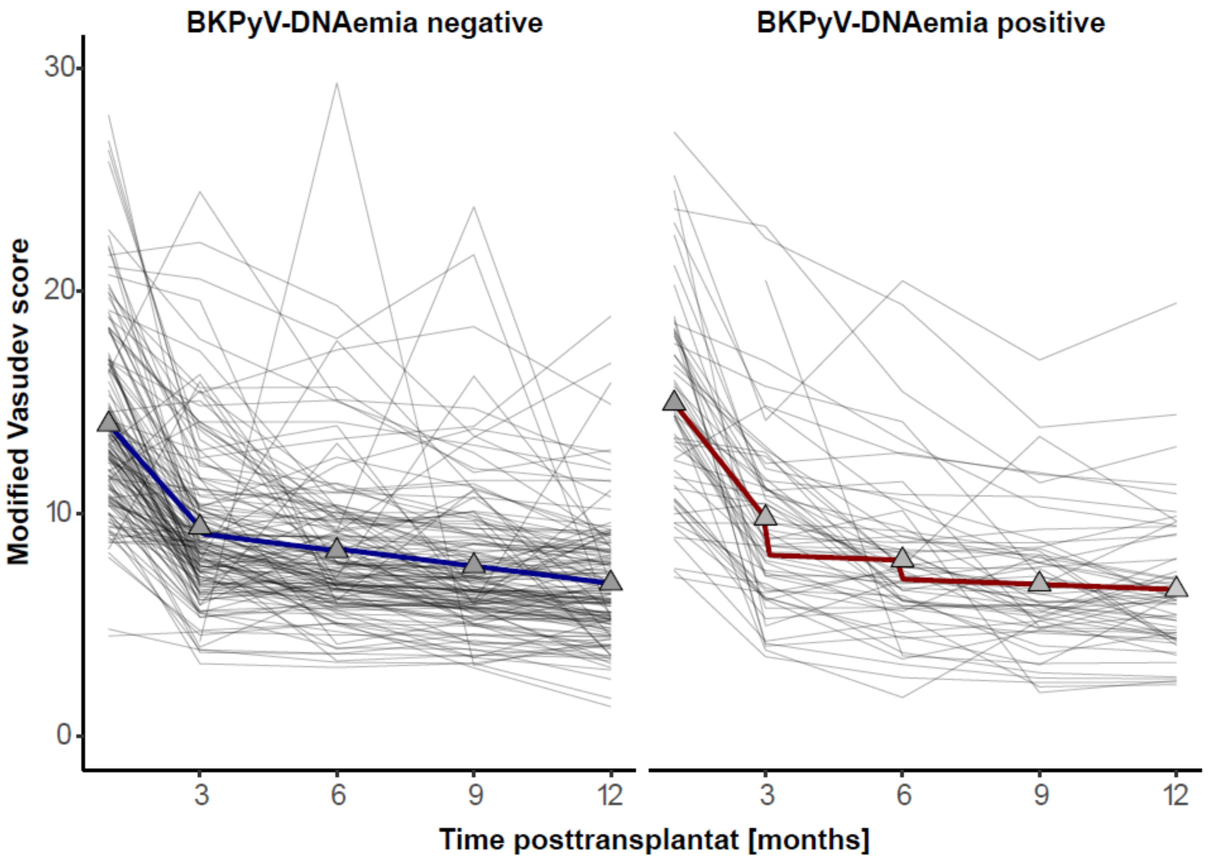

Supplemental Table S1: Modified Vasudev score

Immunosuppressant	Vasudev score: dose per unit (mg/d)	Pediatric score: dose per unit (mg/m²·d)	Immunosuppressive unit
Tacrolimus	2	1.2	1
Cyclosporine A	100	58	1
Sirolimus	2	1.2	1
Mycophenolate Mofetil	500	290	1
Azathioprine	100	58	1
Prednisone equivalent	5	2.9	1

The pediatric score, modified according to Vasudev et al. for assessment of the overall immunosuppressive load, was calculated by adjustment of the adult score to a body surface area (BSA) of 1.0 m², assuming an adult BSA of 1.73 m². Induction therapy with an interleukin-2 receptor antagonist and anti-rejection treatment with steroid pulse therapy were included in this score and assigned to two immunosuppressive units each.

Supplemental Table S 2 **Risk factors analysis for graft function deterioration (eGFR < 30 mL/min·1.73 m² or an eGFR decline ≥ 50% of baseline) in the subgroup of patients without TCMR during BKPyV-DNAemia (n=185).**

	Unadjusted HR (95% CI)	P value	Adjusted HR (95% CI)	P value
**Risk factors for eGFR loss**				
**Entire cohort**				
BKPyV-DNAemia#	2.94 (1.32 – 6.54)	0.008	4.05 (1.68 – 9.81)	0.002
TCMR#	2.45 (1.15 – 5.40)	0.026	2.43 (1.09 – 5.41)	0.029
Higher donor age (years)	1.03 (1.00 – 1.07)	0.022	1.03 (1.01 – 1.06)	0.034
Re-transplantation	4.95 (1.96 – 12.5)	0.000	3.69 (1.22 – 11.1)	0.021
dnDSA/ABMR#	2.97 (1.30 – 6.76)	0.010		
**Low immunologic-risk patients**				
BKPyV-DNAemia#	5.83 (2.26 – 15.0)	0.000	6.37 (2.46 - 16.5)	0.000
TCMR#	2.32 (0.94 – 5.74)	0.042	2.71 (1.09 - 6.73)	0.031

BKPyV, BK polyomavirus; dnDSA, *de novo* donor-specific antibodies; ABMR, antibody-mediated rejection; eGFR, estimated glomerular filtration rate; TCMR, T cell-mediated rejection; #variable included as time-dependent variable in the cox regression model
